# Wearables in the SARS-CoV-2 Pandemic: What Are They Good for?

**DOI:** 10.2196/25137

**Published:** 2020-12-22

**Authors:** Brinnae Bent, Jessilyn P Dunn

**Affiliations:** 1 Department of Biomedical Engineering Duke University Durham, NC United States; 2 Department of Biostatistics and Bioinformatics Duke University Medical Center Durham, NC United States

**Keywords:** digital medicine, digital health, mHealth, wearables, sensors, validation, pandemic, COVID-19

## Abstract

Recently, companies such as Apple Inc, Fitbit Inc, and Garmin Ltd have released new wearable blood oxygenation measurement technologies. Although the release of these technologies has great potential for generating health-related information, it is important to acknowledge the repercussions of consumer-targeted biometric monitoring technologies (BioMeTs), which in practice, are often used for medical decision making. BioMeTs are bodily connected digital medicine products that process data captured by mobile sensors that use algorithms to generate measures of behavioral and physiological function. These BioMeTs span both general wellness products and medical devices, and consumer-targeted BioMeTs intended for general wellness purposes are not required to undergo a standardized and transparent evaluation process for ensuring their quality and accuracy. The combination of product functionality, marketing, and the circumstances of the global SARS-CoV-2 pandemic have inevitably led to the use of consumer-targeted BioMeTs for reporting health-related measurements to drive medical decision making. In this viewpoint, we urge consumer-targeted BioMeT manufacturers to go beyond the bare minimum requirements described in US Food and Drug Administration guidance when releasing information on wellness BioMeTs. We also explore new methods and incentive systems that may result in a clearer public understanding of the performance and intended use of consumer-targeted BioMeTs.

Recently, several big technology companies have released novel wearables with health functionalities, such as wearables capable of measuring blood oxygenation (SpO_2_). In September 2020, Apple Inc released the Apple Watch 6, which is the first Apple wearable with SpO_2_ monitoring capabilities. This comes on the heels of the Fitbit software update in January 2020, which included SpO_2_ monitoring to existing wearables, and the August 2018 release of the Garmin Vivosmart 4, which is one of the earliest wearable consumer-targeted biometric monitoring technologies (BioMeTs) to monitor SpO_2_ at the wrist, with the reported intent of obtaining fitness measurements at high elevations. Currently, consumer-targeted BioMeTs that are intended for general wellness purposes [1] do not require medical device regulatory oversight. Instead, these BioMeTs fall under the oversight of the Federal Trade Commission Act, which prohibits unfair and deceptive acts or practices in commerce [2]. Accordingly, the majority of consumer-targeted BioMeT manufacturers do not publicly report on the performance of their sensor technologies. With the backdrop of the SARS-CoV-2 pandemic, wearables and other bodily connected sensors that are marketed as consumer-targeted wellness BioMeTs are, in practice, being used for health decision making [3-6]. For example, during the recent Apple Watch 6 release event, the new SpO_2_ functionality was referred to as a “health sensor” multiple times by company executives and mentioned in the context of SARS-CoV-2 detection and studies on asthma, heart failure, and influenza [7-9]. The transparency of consumer-targeted BioMeT performance is desperately needed to avoid the misinterpretation of data or improper use, which will ultimately undermine public perception and trust in these products. We propose the following 3 recommendations (Textbox 1): (1) consumer-targeted BioMeT companies should state the intended use of the product for consumers, patients, medical practitioners, and researchers, rather than stating what the products are not intended to be used for (ie, medical decision making); (2) consumer-targeted BioMeT companies should clarify how the data should be interpreted and move qualifying statements about how the products are not intended for health purposes from the fine print to the headlines; and (3) we advocate for clarity surrounding the performance of consumer-targeted BioMeTs to increase the trustworthiness of measurements from these products (Figure 1).

**Recommendations for the transparency of consumer-targeted biometric monitoring technology performance.**

**Recommendations**

- State the use of the product for consumers, patients, medical practitioners, and researchers, rather than stating what the products are not intended to be used for.
- Clarify how data should be interpreted by moving qualifying statements about how the products are not intended for health purposes from the fine print to the headlines.
- Clarify the performance of consumer-targeted biometric monitoring technologies to increase the trustworthiness of measurements from these products. 

**Figure 1:**
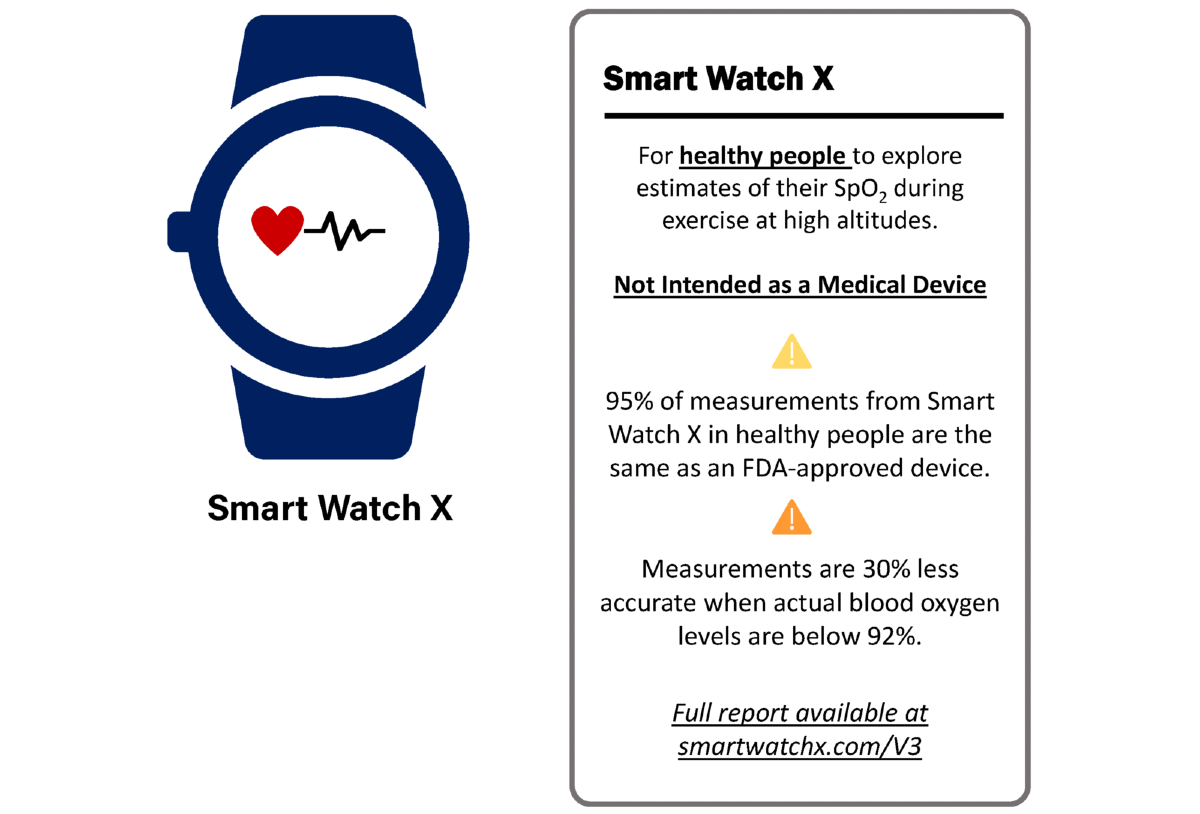
Example of a BioMeT transparency label. We recommend a consumer-targeted BioMeT transparency label that would accompany all product marketing materials, packaging, and mobile applications. The transparency of the performance and intended use of consumer-targeted BioMeTs will cultivate trust and encourage the expansion of BioMeT fit-for-purpose use. BioMeT: biometric monitoring technology.

With the SARS-CoV-2 pandemic, the desire to use consumer-targeted BioMeTs to monitor potential symptoms of infection is understandable, because monitoring for signs of infection at home may reduce anxiety and increase one’s confidence in their health status (ie, healthy or sick). The danger however is that many people are improperly using consumer-targeted BioMeTs to monitor for signs of illness and relying too heavily on data that does not have a sufficient evidence base, as there is no oversight by medical professionals [10,11]. Furthermore, it has become increasingly common for a single product to have differentially regulated features, which only adds to the confusion. For example, the Apple Watch received Food and Drug Administration (FDA) clearance for detecting irregular heart rhythms by using the product’s electrocardiogram sensor. However, the other sensors on the Apple Watch, including the optical heart rate sensor, are unregulated [12]. It can be challenging for consumers to understand which sensors are regulated. With regard to the Apple Watch, heart rate is monitored with 2 different sensors, but only 1 of the sensors is regulated by the FDA. This challenge can be addressed through our first recommendation, which is to change the status quo from manufacturers listing what the product is not intended for in the fine print to manufacturers clearly stating the product’s intended use and specifying the general wellness category based on the FDA guidance document list [1]. For example, Garmin has set a clear target for use, which is evaluating fitness at high altitudes for mountain climbers, hikers, and runners. Other consumer-targeted BioMeT manufacturers have been less transparent about their products’ target for use, resulting in confusion about the intended use for consumers, patients, clinicians, and researchers.

BioMeT users should understand the product’s intended purpose, know about the product’s limitations, and adhere to the instructions for wear to ensure that measurements are interpreted correctly, while also minding the effects of noise, errors, and biological variability on measurements. To support this, information should be provided in an easy-to-find and easy-to-digest format. Recently, researchers at Elektra Labs and the Digital Medicine Society have proposed the use of a “connected sensor label” (ie, similar to a nutrition facts label) to report on the objective measures of a BioMeT’s validation, usability, utility, security, and data governance components [6]. To support transparent validation, the group also developed the V3 Framework, which is a systematic assessment tool for BioMeT performance [13], and very recently published evaluation criteria for using BioMeTs to monitor vital signs during the SARS-CoV-2 pandemic [14]. Perhaps the most integral component of the V3 framework is reporting results in a standardized and transparent manner [6,13,15-17]. These protocols and findings are “key tools for documenting scientific evidence needed to draw inferences on whether a technology is fit-for-purpose for the intended use and context of use” [13].

We can envision several possible scenarios to support and incentivize the open evaluation and reporting of consumer-targeted BioMeT performance (Textbox 2). Manufacturers of consumer-targeted BioMeTs are incentivized by consumer demand, and they can benefit from releasing results that have already been collected through internal product testing to build trust in their products and differentiate their products from those of their competitors. Independent third parties, including research laboratories, consumer groups, and professional societies, can also evaluate and report on the accuracy and quality of BioMeT measurements compared to reference standards. This work can be funded as an extension of the 21st Century Cures Act [18] to support the translation of these products into practice. However, while these practices are not intended to fall under FDA oversight, there is a possibility that these practices could be performed in line with the goal of the new FDA Digital Health Center of Excellence, which is to innovate regulatory approaches for providing efficient and the least burdensome oversight, while also meeting the FDA standards for safe and effective products [19]. The transparency of the performance and intended use of consumer-targeted BioMeTs will cultivate trust and encourage the expansion of BioMeT fit-for-purpose use.

**Nonexclusive scenarios to support and incentivize the open evaluation and reporting of consumer-targeted biometric monitoring technology performance.**

**Incentives for consumer-targeted biometric monitoring technology manufacturers releasing results that have already been collected through internal product testing**

- Consumer demand
- Cultivating trust
- Differentiating their products from competitor products

**Incentives for independent third parties (ie, research laboratories, consumer groups, and professional societies) evaluating and reporting on the accuracy and quality of biometric monitoring technology measurements compared to reference standards**

- Impartial research
- Expanding biometric monitoring technology fit-for-purpose use
- Publication/exposure
- Potential funding by extension of the 21st Century Cures Act

**Incentives for obtaining new regulatory definitions through the Food and Drug Administration Digital Health Center of Excellence**

- Innovating regulatory approaches
- Providing efficient and the least burdensome oversight

## Abbreviations

BioMeT: biometric monitoring technology
FDA: Food and Drug Administration
SpO_2_: blood oxygenation
